# Impact of Frailty Risk on Adverse Outcomes after Traumatic Brain Injury: A Historical Cohort Study

**DOI:** 10.3390/jcm11237064

**Published:** 2022-11-29

**Authors:** Yoshinori Yamamoto, Shinsuke Hori, Kenta Ushida, Yuka Shirai, Miho Shimizu, Yuki Kato, Akio Shimizu, Ryo Momosaki

**Affiliations:** 1 Department of Rehabilitation Medicine, Mie University Graduate School of Medicine, Tsu 514-8507, Japan; 2 Department of Rehabilitation, Mie University Hospital, Tsu 514-8507, Japan; 3 Department of Nutrition, Hamamatsu Medicine University Hospital, Hamamatsu 431-3192, Japan; 4 Department of Health Science, Faculty of Health and Human Development, The University of Nagano, Nagano 380-8525, Japan

**Keywords:** traumatic brain injury, Hospital Frailty Risk Score, frailty, historical cohort study

## Abstract

We evaluated the utility of the Hospital Frailty Risk Score (HFRS) as a predictor of adverse events after hospitalization in a retrospective analysis of traumatic brain injury (TBI). This historical cohort study analyzed the data of patients hospitalized with TBI between April 2014 and August 2020 who were registered in the JMDC database. We used HFRS to classify the patients into the low- (HFRS < 5), intermediate- (HFRS5-15), and high- (HFRS > 15)-frailty risk groups. Outcomes were the length of hospital stay, the number of patients with Barthel Index score ≥ 95 on, Barthel Index gain, and in-hospital death. We used logistic and linear regression analyses to estimate the association between HFRS and outcome in TBI. We included 18,065 patients with TBI (mean age: 71.8 years). Among these patients, 10,139 (56.1%) were in the low-frailty risk group, 7388 (40.9%) were in the intermediate-frailty risk group, and 538 (3.0%) were in the high-frailty risk group. The intermediate- and high-frailty risk groups were characterized by longer hospital stays than the low-frailty risk group (intermediate-frailty risk group: coefficient 1.952, 95%; confidence interval (CI): 1.117–2.786; high-frailty risk group: coefficient 5.770; 95% CI: 3.160–8.379). The intermediate- and high-frailty risk groups were negatively associated with a Barthel Index score ≥ 95 on discharge (intermediate-frailty risk group: odds ratio 0.645; 95% CI: 0.595–0.699; high-frailty risk group: odds ratio 0.221; 95% CI: 0.157–0.311) and Barthel Index gain (intermediate-frailty risk group: coefficient −4.868, 95% CI: −5.599–−3.773; high-frailty risk group: coefficient −19.596, 95% CI: −22.242–−16.714). The intermediate- and high-frailty risk groups were not associated with in-hospital deaths (intermediate-frailty risk group: odds ratio 0.901; 95% CI: 0.766–1.061; high-frailty risk group: odds ratio 0.707; 95% CI: 0.459–1.091). We found that HFRS could predict adverse outcomes during hospitalization in TBI patients.

## 1. Introduction

Traumatic brain injury (TBI) is one of the most severe types of injury in terms of both morbidity and long-term impact on survivors [1,2]. According to the Centers for Disease Control and Prevention, there were approximately 223,135 TBI-related hospitalizations in 2019 and 64,362 TBI-related deaths in 2020 [3]. TBI-related mortality rates increased over the ten years from 2008 to 2017 [3]. Patients with severe TBI could have long-term or life-long disabilities [3]. TBI results in long-term societal costs, and initiatives to improve prognosis and reduce societal costs after TBI have been reported [2,4].

TBI is closely associated with frailty [5]. TBI can be caused by falls, traffic accidents, and other injuries, with falls causing approximately half of all TBIs [6]. The Centers for Disease Control and Prevention reported that TBI-related emergency hospitalizations in 2014 were the highest among seniors aged ≥ 75 years [6]. Frailty is a condition in which motor and cognitive functions decline with age, and falls related to frailty are particularly common in the older population [5,7]. Additionally, frailty has several characteristics that affect TBI management and outcomes, including multimorbidity and polypharmacy [8].

Recently, the Hospital Frailty Risk Score (HFRS), based on the 10th revision of the International Classification of Diseases (ICD-10) diagnostic codes, has been developed and reported to be useful for identifying patients at high risk for adverse outcomes [9]. HFRS is calculated based on 109 ICD-10 codes, with specific weights applied to each code. HFRS could be implemented in most hospital information systems with less time and at a low cost [9,10]. HFRS has been reported to predict frailty and adverse outcomes in the general population [9,10].

Assessment of the risk of frailty in patients with TBI could help predict and prevent adverse outcomes. Several studies have predicted prognosis using the frailty scale for TBI [11,12,13,14]. Some studies used frailty assessment scales based on medication and biochemical examination data [11,12,13], which are difficult to calculate automatically. One study [14] assessed frailty based on 11 comorbidities; however, this was reported to be less accurate at predicting adverse events than a frailty assessment scale based on a larger number of comorbidities [15]. Therefore, we focused on HFRS, which can be calculated automatically in the presence of several comorbidities. To the best of our knowledge, HFRS has not been investigated for its potential to predict adverse outcomes in TBI. We hypothesized that patients with TBI at a higher risk of frailty, as calculated by HFRS, would have longer hospital stays, more deaths, and lower activities of daily living (ADL) independence than patients with TBI at a lower risk of frailty. The purpose of this cohort study was to evaluate the utility of HFRS as a predictor of adverse events after TBI.

## 2. Materials and Methods

### 2.1. Ethical Considerations

This study used data from a hospital-based database created by the JMDC. The requirement for obtaining patient informed consent was waived because the data provided by JMDC was de-identified and unlinked to personal information. This database is generally available; hence, the ethics committee of Mie University determined that no ethical review was required.

### 2.2. Data Source

This retrospective cohort study is observational research using the JMDC database. The JMDC database has been accumulating medical fee data from multiple health insurance societies since 2005 [16,17]. The database includes individual health insurance claims from >60 insurers: the cumulative population size amounts to approximately 14 million people (as of February 2022) [18]. The database includes Diagnostic Procedure Combination data, which was introduced to Japan in 2003 as a system to pay for medical care [19]. The Diagnostic Procedure Combination database is a national database of acute inpatient admissions in Japan. The data include sex, age, ICD-10 diagnostic codes, surgical information, length of stay (LOS), Barthel Index (BI), and other information [20].

### 2.3. Study Population

Disease diagnosis data in the Diagnosis Procedure Combination database are classified with ICD-10 codes recorded by the attending physician. This study analyzed the data for the period from April 2014 to August 2020 from patients hospitalized due to TBI (ICD-10 code S60). We excluded patients with missing Japan coma scale (JCS) score data at admission, BI at discharge, and BI at admission.

### 2.4. Calculation of HFRS

We calculated HFRS at the time of hospitalization for patients admitted with a diagnosis of TBI [9]. This Frailty index was developed and endorsed using data from 1 million patients aged > 74 years [9]. HFRS is calculated by summing points assigned to each of the 109 ICD-10 codes. HFRS in this study is calculated from the comorbidities of each patient at admission. HFRS comprises 31 comorbidities, including dementia, delirium, neuropathy, pneumonia, urinary tract disorders, hypotension, fractures, and motor dysfunction [9]. HFRS assigns different points to each code, with higher scores indicating greater frailty risk [9]. In HFRS, the cutoff point for frailty is reported to be ≥ 5 [9]. We used HFRS to classify patients into the low- (HFRS < 5), intermediate- (HFRS 5–15), and high- (HFRS > 15) frailty risk groups.

### 2.5. Variables and Outcomes

The variables used in this study were as follows: sex, age, body mass index, type of TBI (diffuse TBI S062, focal TBI S063, epidural hemorrhage S064, traumatic subdural hemorrhage S065, traumatic subarachnoid hemorrhage S066, and other injury types), neurosurgical surgery on admission, ventilator use on admission, intensive care unit management on admission, JCS score at admission, BI at admission, inpatient rehabilitation services received, number of drugs taken during hospitalization, the mean number of complications occurring during hospitalization, number of beds, and year of admission. Surgery includes craniotomy. The JCS is a widely used tool in Japan to assess the level of consciousness, which is classified as follows: alert (0), dull (1-digit code: 1, 2, 3), somnolence (2-digit code: 10, 20, 30), and coma (3-digit code: 100, 200, 300) [21]. The BI is used to assess a patient’s ability to perform ADL; a higher BI score indicates greater independence in ADL [22].

In this study, the outcomes were LOS, BI score ≥ 95 on, BI gain, and hospital death. BI gain was determined as the BI score at discharge less the BI score on admission. BI is considered fully independent, with BI score ≥ 95 on discharge [22].

### 2.6. Statistical Analysis

We compared the outcomes between the low-, intermediate-, and high-frailty risk groups. The data were expressed as absolute values and percentages for categorical data and mean ± standard deviation for continuous data. The χ^2^ test was used to determine differences among the three groups. Differences in the frailty risk among the three groups were analyzed using the one-way analysis of variance. We performed multivariate logistic and multiple linear regression analyses to adjust for confounding factors. The covariates adjusted were sex, age, body mass index, type of TBI, surgery on admission, ventilator use on admission, intensive care unit management on admission, JCS score at admission, BI at admission, inpatient rehabilitation services received, number of drugs administered during hospitalization, number of complications that occurred during hospitalization, number of beds, and year of admission. Statistical analyses were performed using SPSS software (version 25.0; IBM Japan, Tokyo, Japan). Statistical significance was set at *p* < 0.05.

## 3. Results

**Figure 1:**
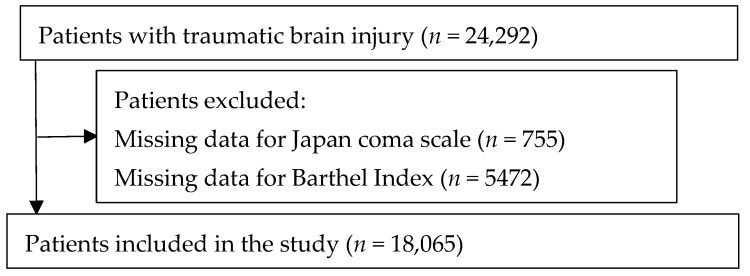
Patient selection.

We included 18,065 patients who were hospitalized due to TBI, who did not have missing data on JCS score at admission, BI at admission, and BI at discharge (Figure 1). Patients were classified into the low- (10,139 (56.1%)), intermediate- (7388 (40.9%)), and high- (538 (3.0%)) frailty risk groups based on HFRS.

Table 1 shows the characteristics of the study participants. The high-frailty risk group had more women, a lower JCS score and BI on admission, and a higher proportion of patients aged > 75 years than the low- and intermediate-frailty risk groups.

Table 2 shows the results of outcome comparisons among the groups. The frailty risk was significantly associated with LOS, the number of patients with BI score ≥ 95 on discharge, BI gain, and the number of in-hospital deaths.

Table 3 shows the results of the multiple linear regression analyses on HFRS. The intermediate- and high-frailty risk groups were characterized by longer hospital stays than the low-frailty risk group (intermediate-frailty risk group: coefficient 1.952, 95% CI: 1.117–2.786; high-frailty risk group: coefficient 5.770, 95% CI: 3.160–8.379). The intermediate- and high-frailty risk groups were negatively associated with BI gain (intermediate-frailty risk group: coefficient −4.868, 95% CI: −5.599–−3.773; high-frailty risk group: coefficient −19.596, 95% CI: −22.242–−16.714).

Table 4 shows the results of the logistic regression analysis on HFRS. The intermediate- and high-frailty risk groups were negatively associated with a BI score ≥ 95 on discharge (intermediate-frailty risk group: odds ratio 0.645; 95% CI: 0.595−0.699; high-frailty risk group: odds ratio 0.221; 95% CI: 0.157−0.311). The intermediate- and high-frailty risk groups were not significantly associated with in-hospital death (intermediate-frailty risk group: odds ratio 0.901; 95% CI: 0.766−1.061; high-frailty risk group: odds ratio 0.707; 95% CI: 0.459−1.091).

## 4. Discussion

This study investigated the association of HFRS assessed using ICD-10 codes with adverse events and functional outcomes in patients with TBI, using nationwide data from Japan. The results indicated that patients with a higher frailty risk had a longer hospital stay duration and lower ability to perform ADL during hospitalization.

High frailty risk was positively associated with the length of the hospital stay. HFRS assesses frailty risk based on a weighted score of comorbidities with a certain number of points assigned for each ICD-10 code [9]. Gilbert et al. [9] reported that, among patients aged > 75 years admitted to an acute care hospital, those with a high frailty risk had a nearly two-fold increase in 30-day mortality and a six-fold increase in the risk of long-term hospitalization compared to those with a low frailty risk. Subsequent studies have examined the association between HFRS and adverse events in patients admitted to medical facilities [23] with stroke/transient ischemic attack [24], hip fracture surgery [25], total hip and knee arthroplasty [26], osteoarthritis [27], chronic obstructive pulmonary disease [28], and heart failure [29]. These reports stated that HFRS could predict adverse events during hospitalization. Similar to previous studies, we found that HFRS could predict prolonged LOS in patients with TBI. Patients with more comorbidities had a higher risk for mortality and severe disease [30]. Frailty was also considered a condition with a high risk of adverse outcomes due to the loss of function in multiple organs, such as the brain, endocrine system, immune system, and skeletal muscles [7]. Therefore, HFRS using comorbidities could be a useful tool for predicting adverse events in patients with TBI.

The high frailty risk was negatively associated with the number of patients with BI score ≥ 95 on discharge and BI gain. HFRS was suggested to predict functional outcomes, e.g., ADL, in TBI patients. High frailty risk was also not associated with in-hospital death. In HFRS, there is a greater weighting of ICD-10 codes for cerebrovascular, motor, gait, and cognitive disabilities [9]. HFRS includes diseases based on ICD-10 codes that affect functional outcomes [9,28]. Thus, patients at a higher risk of frailty are more likely to present with comorbidities that affect functional recovery more. HFRS has fewer comorbidities that are directly related to death than functional impairment and has less accuracy in predicting death [28,31]. Therefore, HFRS could be a useful predictive tool for functional prognoses, such as ADL, rather than death. In contrast, in a study that followed up patients with TBI for 1 year, death was found to be associated with frailty [32]. A long-term follow-up in the present study might have increased the number of deaths as well as the statistical power to detect significant differences.

Patients at a intermediate-, and high risk of frailty accounted for about half of the patients with TBI (43.9%). The percentage of TBI patients with frailty was approximately 40% using the mFI-11 [14] and Groningen Frailty Indicator [13]. In contrast, patients with frailty alongside other conditions assessed using HFRS (total hip arthroplasty/total knee arthroplasty [21] and spinal surgery [27]) included fewer than 15% of patients at a moderate to severe frailty risk. Falls in the older population are closely related to frailty, an age-related decline in motor and cognitive function [5,6]. Factors contributing to falls include age, sex, gait disturbance, and neurological and cognitive impairments [5,7]. Thus, patients with TBI may have been affected by frailty even before the injury. TBI is also likely to result in life-long physical, cognitive, and psychosocial functional impairments [33,34,35]. HFRS has many items associated with TBI (cerebrovascular disease, motor impairment, gait disturbance, cognitive impairment, open or superficial head injury, delirium, emotional status, and so on), which may have contributed to the high proportion of patients at a high risk of frailty.

This study has several strengths. Several comorbidity scores have been previously developed, including the Charlson Comorbidity Index and Elixhauser Comorbidity Index [36,37]. HFRS includes cognitive impairment, delirium, disorientation, falls, mobility impairments, dependence syndrome, urinary incontinence, and difficulties in managing life that have not received attention in the Charlson and Elixhauser Comorbidity Indexes [9]. HFRS includes more comorbidities related to injury in TBI than other comorbidity indices [9]. Therefore, compared to other comorbidity indices, HFRS could better detect functional adverse events at admission in TBI patients. Another strength is that HFRS can be automatically implemented in in-hospital information systems [9]. Because ICD-10 codes are routinely recorded electronically, HFRS data for TBI can be automatically embedded in in-hospital electronic medical records. HFRS could be made available automatically, potentially reducing patient burden by predicting adverse events.

This study has several limitations that are common to database studies. The analysis is a historical cohort study, and data acquisition was limited to data available from the hospital information system. Therefore, we were unable to obtain long-term follow-up data or detailed data on parameters such as walking ability, muscle strength, or Glasgow Coma Scale score. However, a high correlation between the JCS and Glasgow Coma Scale scores has been reported [22], and we believe that we were able to adequately assess disease severity. In addition, the accuracy of this data could be somewhat problematic because HFRS used in this study was based on ICD-10 codes in the medical information system. In general, studies using reimbursement databases reported problems with data reliability owing to coding errors, inappropriate coding, and poor documentation [38,39]. However, the criterion-related validity of medical records based on ICD-10 codes has been validated in Japan [20]. Therefore, there may have been minimal bias due to coding inaccuracy.

## 5. Conclusions

This study suggests that HFRS could predict adverse outcomes for TBI in advance. Patients at a higher risk for frailty were associated with longer hospital stays, a lower BI gain, and a lower percentage of patients with BI score ≥ 95 on discharge. This finding supports the utility of HFRS as a predictor of adverse events after hospitalization due to TBI.

## Figures and Tables

**Table 1:** Characteristics of the study participants.

	Low-Frailty Risk Group (Hospital Frailty Risk Score < 5)	Intermediate-Frailty Risk Group (Hospital Frailty Risk Score 5–15)	High-Frailty Risk Group (Hospital Frailty Risk Score > 15)	*p*-Value
Number of patients, *n* (%)	10,139 (56.1)	7388 (40.9)	538 (3.0)	
Female sex, *n* (%)	3607 (35.6)	2944 (39.8)	292 (54.3)	<0.001
Age, years, *n* (%)				<0.001
-<65	2444 (24.1)	1384 (18.7)	8 (1.5)	
-65–74	2214 (21.8)	1397 (18.9)	34 (6.3)	
-75–89	4418 (43.6)	3608 (48.8)	375 (69.7)	
-≥90	1063 (10.5)	999 (13.5)	121 (22.5)	
Body mass index, *n* (%)				<0.001
-<18.5	1742 (17.2)	1498 (20.3)	158 (29.4)	
-18.5–25.0	5517 (54.4)	3861 (52.3)	241 (44.8)	
-≥25.0	1680 (16.6)	1082 (14.6)	55 (10.2)	
-Missing	1200 (11.8)	947 (12.8)	84 (15.6)	
Injury type, *n* (%)				<0.001
-Diffuse traumatic brain injury	907 (8.9)	712 (9.6)	42 (7.8)	
-Focal traumatic brain injury	301 (3.0)	208 (2.8)	12 (2.2)	
-Epidural hemorrhage	363 (3.6)	261 (3.5)	13 (2.4)	
-Traumatic subdural hemorrhage	5549 (54.7)	3671 (49.7)	340 (63.2)	
-Traumatic subarachnoid hemorrhage	1923 (19.0)	1597 (21.6)	98 (18.2)	
-Other injury type	1096 (10.8)	939 (12.7)	33 (6.1)	
Neurosurgical procedure on admission, *n* (%)	3248 (32.0)	1706 (23.1)	169 (31.4)	<0.001
Ventilator on admission, *n* (%)	340 (3.4)	495 (6.7)	30 (5.6)	<0.001
Intensive care unit management on admission, *n* (%)	929 (9.2)	925 (12.5)	67 (12.5)	<0.001
Japan coma scale at admission, *n* (%)				<0.001
-0	4960 (48.9)	2545 (34.4)	118 (21.9)	
-1–3	3934 (38.8)	3438 (46.5)	304 (56.5)	
-10–30	659 (6.5)	765 (10.4)	75 (13.9)	
-100–300	586 (5.8)	640 (8.7)	41 (7.6)	
Barthel index at admission, mean (SD)	47.7 ± 40.8	33.9 ± 38.1	19.3 ± 29.7	<0.001
Received inpatient rehabilitation services, *n* (%)	5150 (50.8)	4489 (60.8)	423 (78.6)	<0.001
Number of drugs during hospitalization, mean (SD)	4.4 (4.5)	5.9 (5.2)	7.5 (5.3)	0.004
Number of complications occurring during hospitalization, mean (SD)	0.5 (1.0)	1.5 (1.8)	2.4 (2.3)	<0.001
Number of beds, *n* (%)				<0.001
-20–99	186 (1.8)	98 (1.3)	8 (1.5)	
-100–199	2436 (24.0)	1334 (18.1)	131 (24.3)	
-200–299	1650 (16.3)	1196 (16.2)	120 (22.3)	
-300–499	2987 (29.5)	2664 (36.1)	165 (30.7)	
-≥500	2880 (28.4)	2096 (28.4)	114 (21.2)	
Year of admission, *n* (%)				<0.001
-2014	858 (8.5)	534 (7.2)	24 (4.5)	
-2015	1308 (12.9)	810 (11.0)	36 (6.7)	
-2016	1498 (14.8)	1148 (15.5)	77 (14.3)	
-2017	1650 (16.3)	1292 (17.5)	108 (20.1)	
-2018	2004 (19.8)	1412 (19.1)	119 (22.1)	
-2019	1908 (18.8)	1441 (19.5)	120 (22.3)	
-2020	913 (9.0)	751 (10.2)	54 (10.0)	

**Table 2:** Comparison of Hospital Frailty Risk Score and outcomes between the low-, intermediate-, and high-frailty risk groups.

	Low-Frailty Risk Group (Hospital Frailty Risk Score < 5)	Intermediate-Frailty Risk Group (Hospital Frailty Risk Score 5–15)	High-Frailty Risk Group (Hospital Frailty Risk Score > 15)	*p*-Value
Length of hospital stay, Mean ± SD	16.6 ± 27.9	25.0 ± 35.9	36.1 ± 42.9	<0.001
Barthel Index score ≥ 95 on discharge, *n* (%)	6034 (59.5)	2950 (39.9)	59 (11.0)	<0.001
Barthel index gain, Mean ± SD	26.9 ± 37.4	25.1 ±38.2	14.0 ± 30.5	<0.001
Death in hospital, *n* (%)	655 (6.5)	640 (8.7)	40 (7.4)	<0.001

**Table 3:** Multiple linear regression analysis for the length of hospital stay and Barthel Index gain.

Variables	Coefficient	95% Confidence Interval		*p*-Value
		Lower	Upper	
Length of hospital stay				
Low-frailty risk group (reference)	-	-	-	
Intermediate-frailty risk group	1.952	1.117	2.786	<0.001
High-frailty risk group	5.770	3.160	8.379	<0.001
Barthel index gain				
Low-frailty risk group (reference)	-	-	-	
Intermediate-frailty risk group	−4.868	−5.599	−3.773	<0.001
High-frailty risk group	−19.596	−22.242	−16.714	<0.001

Models adjusted for sex, age, body mass index, injury type, neurosurgical procedure on admission, ventilator use on admission, intensive care unit management on admission, Japan coma scale score on admission, Barthel Index on admission, inpatient rehabilitation services received, number of drugs administered during hospitalization, number of complications that occurred during hospitalization, number of beds, and year of admission.

**Table 4:** Binary logistic regression analysis for in-hospital death and Barthel Index score ≥ 95 on discharge.

Variables	Odds Ratio	95% Confidence Interval		*p*-Value
		Lower	Upper	
Barthel Index score ≥ 95 on discharge				
Low-frailty risk group (reference)	-	-	-	
Intermediate-frailty risk group	0.645	0.595	0.699	<0.001
High-frailty risk group	0.221	0.157	0.311	<0.001
Death in hospital				
Low-frailty risk group (reference)	-	-	-	
Intermediate-frailty risk group	0.901	0.766	1.061	0.690
High-frailty risk group	0.707	0.459	1.091	0.245

Models adjusted for sex, age, body mass index, injury type, neurosurgical procedure on admission, ventilator use on admission, intensive care unit management on admission, Japan coma scale score at admission, Barthel Index at admission, inpatient rehabilitation services received, number of drugs administered during hospitalization, number of complications that occurred during hospitalization, number of beds, and year of admission.

## Data Availability

Data sharing not applicable—no new data generated.
